# Host Decoy Trap (HDT) with cattle odour is highly effective for collection of exophagic malaria vectors

**DOI:** 10.1186/s13071-018-3099-7

**Published:** 2018-10-15

**Authors:** Bernard Abong’o, Xiaoyu Yu, Martin J. Donnelly, Martin Geier, Gabriella Gibson, John Gimnig, Feiko ter Kuile, Neil F. Lobo, Eric Ochomo, Stephen Munga, Maurice Ombok, Aaron Samuels, Stephen J. Torr, Frances M. Hawkes

**Affiliations:** 1 Liverpool School of Tropical Medicine, Pembroke Place, Liverpool, L3 5QA UK; 2 Centre for Global Health Research, Kenya Medical Research Institute, PO Box 1578-40100, Kisumu, Kenya; 3 Abt Associates Inc. PMI-VectorLink Kenya, Whitehouse, Milimani, Kisumu, Ojijo Oteko Road, P.O. Box 895-40123, Kisumu, Kenya; 4 Eck Institute for Global Health, Department of Biological Sciences, University of Notre Dame, Notre Dame, IN 46556 USA; 5 Biogents AB GmbH, Regensburg, Germany; 6 Natural Resources Institute, University of Greenwich at Medway, Chatham Maritime, Kent, ME4 4TB UK; 7 Division of Parasitic Diseases and Malaria, Center for Global Health, Centers for Disease Control and Prevention, Atlanta, GA 30333 USA; 8 Centers for Disease Control and Prevention, Kisian Campus, Off Busia Road, P O Box 1578, Kisumu, 40100 Kenya

**Keywords:** *Anopheles*, *An. arabiensis*, *An. gambiae* (*s.s*.), Vector behaviour, Host, Odour, Mosquito trap, Exophily

## Abstract

**Background:**

As currently implemented, malaria vector surveillance in sub-Saharan Africa targets endophagic and endophilic mosquitoes, leaving exophagic (outdoor blood-feeding) mosquitoes underrepresented. We evaluated the recently developed host decoy trap (HDT) and compared it to the gold standard, human landing catch (HLC), in a 3 × 3 Latin square study design outdoors in western Kenya. HLCs are considered to represent the natural range of *Anopheles* biting-behaviour compared to other sampling tools, and therefore, in principle, provide the most reliable profile of the biting population transmitting malaria. The HDT incorporates the main host stimuli that attract blood-meal seeking mosquitoes and can be baited with the odours of live hosts.

**Results:**

Numbers and species diversity of trapped mosquitoes varied significantly between HLCs and HDTs baited with human (HDT-H) or cattle (HDT-C) odour, revealing important differences in behaviour of *Anopheles* species. In the main study in Kisian, the HDT-C collected a nightly mean of 43.2 (95% CI: 26.7–69.8) *Anopheles*, compared to 5.8 (95% CI: 4.1–8.2) in HLC, while HDT-H collected 0.97 (95% CI: 0.4–2.1), significantly fewer than the HLC. Significantly higher proportions of *An. arabiensis* were caught in HDT-Cs (0.94 ± 0.01; SE) and HDT-Hs (0.76 ± 0.09; SE) than in HLCs (0.45 ± 0.05; SE) per trapping night. The proportion of *An. gambiae* (*s.s*.) was highest in HLC (0.55 ± 0.05; SE) followed by HDT-H (0.20 ± 0.09; SE) and least in HDT-C (0.06 ± 0.01; SE). An unbaited HDT placed beside locales where cattle are usually corralled overnight caught mostly *An. arabiensis* with proportions of 0.97 ± 0.02 and 0.80 ± 0.2 relative to the total anopheline catch in the presence and absence of cattle, respectively. A mean of 10.4 (95% CI: 2.0–55.0) *Anopheles*/night were trapped near cattle, compared to 0.4 (95% CI: 0.1–1.7) in unbaited HDT away from hosts.

**Conclusions:**

The capability of HDTs to combine host odours, heat and visual stimuli to simulate a host provides the basis of a system to sample human- and cattle-biting mosquitoes. HDT-C is particularly effective for collecting *An. arabiensis* outdoors. The HDT offers the prospect of a system to monitor and potentially control *An. arabiensis* and other outdoor-biting mosquitoes more effectively.

## Background

Sustained use of long-lasting insecticide treated nets (LLINs) and indoor residual spraying (IRS) have reduced malaria infection prevalence by half between 2000 and 2015, with LLINs and IRS contributing an estimated 68 and 11% of this decline, respectively [1]. Significant changes in vector populations have also been observed with sustained implementation of LLINs [2–4]. Both interventions, however, are limited to indoor application and are therefore more effective against indoor resting (‘endophilic’) and indoor feeding (‘endophagic’) mosquitoes and less so against those that feed and rest outdoors, such as *An. arabiensis* and *An. culicifacies* [5]. Sustained use of LLINs and IRS may also select for outdoor resting (‘exophily’) and feeding (‘exophagy’) in mosquito populations [6–8], day-time feeding [9] and a shift towards non-human hosts (‘zoophagy’), such as cattle [10]. Mosquito populations that feed or rest outdoors may play an important role in the maintenance of malaria transmission after implementation of LLINs or IRS [7]. Accordingly, there is a pressing need for better methods to control and monitor these species.

Methods for sampling adult mosquitoes often exploit host-oriented behaviour. For instance, use of the human landing catch (HLC) or placement of CDC-light traps adjacent to a human under a bednet [11] rely on the attraction of mosquitoes to their host [12–14]. Hitherto, research to develop devices to attract malaria mosquitoes has focused largely on human odours. Identification of the chemicals present in human odour has led to the development of blends of artificial odours [15], which have been used with Mosquito Magnet® X (MMX) [16] and Suna [17] traps to sample and control [18] *An. gambiae* (*s.l.*). However, the design of some of these traps, such as light traps, are dependent on actively aspirating mosquitoes *via* a fan, thereby limiting catch efficacy, as odours induce only part of the behavioral sequence that leads a mosquito to a host [19]. Artificial odour blends in isolation do not fully mimic the range of physical and visual stimuli that attract mosquitoes to natural hosts, particularly those that most influence their close-range orientation behaviour [20–22].

Laboratory studies have begun to quantify synergistic effects between olfactory, visual and thermal cues on mosquito behaviour during host location [21, 23]. These developments can contribute to more effective ways to measure vector-host contact, particularly in outdoor environments, where HLCs remain an important means of sampling, despite exposing collectors to mosquito bites and data quality relying on individual collector skill [22]. A recent study showed that exploitation of the responses of mosquitoes to the heat produced by hosts may be a potent tool for monitoring and controlling outdoor-biting species of mosquito; the host decoy trap (HDT), which combines natural human odour, visual stimuli, and a thermal signature equivalent to the human body, caught between two and tenfold more *An. coluzzii* than a field technician performing HLC outdoors [24], even though *An. coluzzii* is generally considered a primarily endophagic and endophilic species.

In East and Southern Africa, *An. gambiae* (*s.s*.), *An. arabiensis* and *An. funestus* are important vectors of malaria. *Anopheles arabiensis* feeds mostly outdoors on humans and cattle [25–27] while *An. gambiae* (*s.s*.) and *An. funestus* mostly feed indoors on humans [2, 26, 27]. In western Kenya, we tested the relative performance of HDTs baited with either natural human (HDT-H) or cattle (HDT-C) odours against HLC to attract and trap outdoor biting mosquitoes and assessed whether natural host odours might provide an effective basis for systems to monitor and control exophagic and zoophagic vectors of malaria.

## Methods

### Study area

The study was conducted in Kisian village (0.0749°S, 34.6663°E), near the Kenya Medical Research Institute Centre for Global Health Research (KEMRI-CGHR) in Kisumu County, and in Orego village (0.6167°S, 34.55°E), Homa Bay County, western Kenya, in May and June 2017. Western Kenya is malaria endemic with transmission occurring throughout the year. The region has two wet seasons, March to June and October-December, corresponding to periods of highest malaria transmission. Residents are predominantly of Luo ethnic group practicing small-scale mixed crop farming and raising livestock including cattle and goats. *Anopheles funestus*, *An. arabiensis* and *An. gambiae* (*s.s*.) are the main malaria vectors in the study area. The region has high coverage with LLINs (> 85% of households with at least one net) [28].

### Mosquito collection methods

#### Host decoy trap (HDT)

**Fig. 1:**
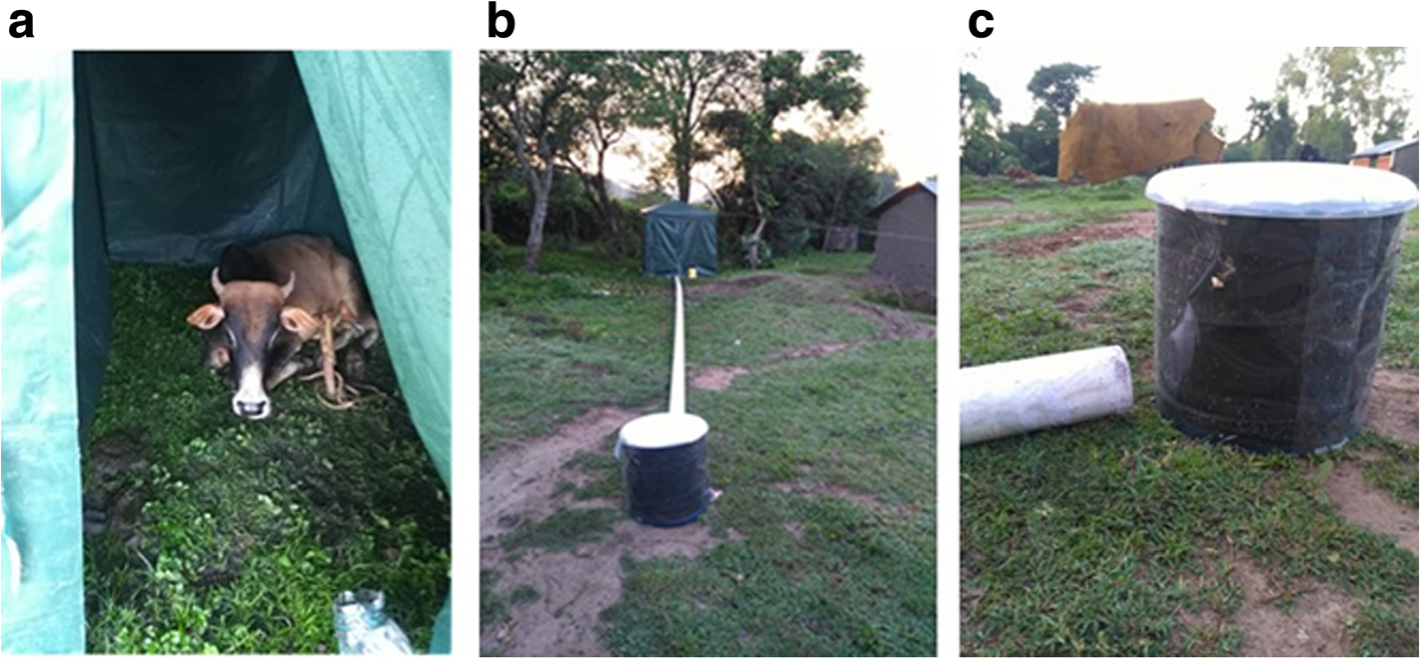
Host Decoy Trap (BG-HDT) set-up. **a** Cow tethered inside tent provides natural host odour and carbon dioxide for baiting HDT. **b** Experimental set-up showing host-occupied tent, PVC pipe (fan inside pipe directs host odour to trap) and HDT. **c** Pipe opening releases host odour within 10 cm of the HDT. Visual stimuli of the dark trap and warmth of water-filled trap induce mosquitoes to land on clear adhesive sheet covering dark surface of the trap

A standardized HDT was manufactured by the University of Greenwich and Biogents AG (BG-HDT version) using the same principles as the prototype described in Hawkes et al. [24]. It consists of a watertight lay-flat plasticized aluminium foil container similar to packets of single-use fruit juice drinks, insulated with layers of polystyrene held in a collapsible cylindrical bucket (height 36 cm, diameter 38 cm), around which a black fabric jacket is secured using hook and eye strips. The watertight bag is filled with ~15 l of water heated to ~80 °C, which is sufficient to maintain surface temperature across the fabric jacket of 30–40 °C for at least 12 hours. The bucket is closed with a transparent polyethylene plastic cover to protect the interior from rain. This unit provides high contrast visual stimuli and human-equivalent thermal stimuli to induce close-range attraction and landing behaviour in host-seeking mosquitoes. A transparent adhesive plastic sheet (FICS film, Barrettine Environmental Health, Bristol, UK) covers the circumference of the trap (Fig. 1a) to catch mosquitoes as they land. In contrast, the original host decoy trap (O-HDT) consisted of metal cooking pot or plastic barrel/container (~40 l), with 15–20 l of hot water. The container was insulated with towelling material to maintain the surface temperature at 30–40 °C. A black fabric “jacket” was sewn to fit over the insulating material to provide a strong visual contrast against the background.

To provide natural host odours, two tents made from canvas supported by a metal frame, each measuring 2.0 m high × 2.0 m square were used to house odour baits (Fig. 1a). One tent was assigned to a cow and another to a human volunteer throughout the study period. Tents were aerated and rotated between the trapping sites each night. A 12V fan (Biogents AG) connected to a 10 m length of PVC tubing (10 cm in diameter) was placed inside the tent (Fig. 1b). The other opening of the tube was covered with untreated mosquito netting and placed ~10 cm from the base of the HDT unit, thus venting host odours from the tent around the trap at approximately 2000 l/min (Fig. 1c). Carbon dioxide produced by both cow and human baited tents was measured at the pipe outlet using a CO_2_ meter (EGM-4, PP Systems, Amesbury, MA, USA). The values were adjusted to consider background levels of CO_2_ (400 ppm). The cow-odour released ~0.6 l/min CO_2_ from the pipe outlet, about three times more than the human-odour released (~0.2 l/min), consistent with an approximately three-fold difference in their weights.

**Fig. 2:**
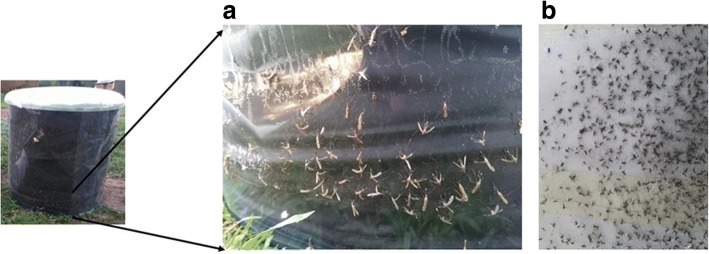
Mosquitoes collected by Host Decoy Traps (HDT). **a** A section of the HDT showing trapped mosquitoes stuck to clear adhesive sheet. **b** Trapped mosquitoes recovered from HDT by removing adhesive sheet from the trap and covering it with a layer of thin plastic food wrap before species identification in the laboratory

In principle, mosquitoes following odour plumes emanating from the end of the PVC tube see the HDT and approach it. They then encounter the warmth of the trap’s surface, whereupon they land and become stuck to the transparent adhesive sheet (Fig. 2a). At the end of the sampling period a thinner plastic sheet of transparent polyethylene wrap (cling film/food wrap) was laid on the surface of the adhesive sheet, sandwiching trapped mosquitoes between the two sheets (Fig. 2b). Using a razorblade, the sheets were cut and removed from the HDT and mosquitoes were later removed from the sheets in the laboratory using Romax Glue Solvent (Barrettine Environmental Health, Bristol, UK).

Whole host odours were used to attract mosquitoes to HDTs. Four cows, each weighing 150–200 kg were used individually to provide natural odours in the experiment. Each cow was used for six consecutive nights before being replaced (Fig. 1a). Eight field assistants working in pairs conducted the experiments, with each pair participating for six consecutive nights before being replaced. The field assistants worked in two shifts (18:00 to 12:00 h and 12:00 to 07:00 h), changing places each night to perform either an outdoor HLC or sleeping in the tent to provide human odour for the HDT-H.

#### Human landing catch (HLC)

Field assistants performing HLCs sat outside at the same locations as the HDT sites, with their trousers folded to knee height and caught mosquitoes landing on their exposed lower limbs using a mouth aspirator. Collections were performed for 45 min and the collectors rested 15 min in each collection hour. Collected mosquitoes were placed in paper cups and were sustained on 10% sugar solution before transportation to the laboratory for analysis.

### Species identification and parasite detection

Mosquitoes were sorted to subfamilies to separate anopheline from culicine species. In each subfamily, mosquitoes were further separated by abdominal status as either fed, unfed, gravid or half gravid. All *Anopheles* mosquitoes were identified morphologically to species [29, 30] and then placed singly in 1.5 ml micro-centrifuge tubes for further laboratory analysis. Species were identified by PCR for *An. gambiae* (*s.l.*) [31] and *An. funestus* (*s.l.*) group [32] and sporozoite rate was determined by enzyme linked immunosorbent assay (ELISA) [33].

### Experiment 1: Comparison of catches from HDTs and HLCs

We investigated the host choices of outdoor-biting malaria vectors using the BG-HDT, baited with either human or cattle odour, and compared these catches with the HLC. Our null hypothesis was that an HLC and the HDTs baited with cow (HDT-C) or human (HDT-H) odour would catch equal numbers of mosquitoes with the same species composition in an outdoor peri-domestic environment. A replicated Latin Square experimental design of collection methods × sites × nights was conducted. Collection sites were 100 m from each other. The experiment was carried out twice, first (May 2017) in Kisian village, Kisumu county, and subsequently (June 2017) in Orego village, Homa Bay county. Collections ran from 18:00 h to 07:00 h for 24 nights in Kisian village and 12 nights in Orego village.

### Experiment 2: Catches from un-baited HDT

In the second experiment, we tested whether mosquitoes would be attracted to an unbaited BG-HDT (i.e. operated without any host odours released from the tent) placed within 5 m of a corralled herd of cattle. The main aim was to determine whether dispersed host odour is sufficient to attract mosquitoes close enough to the HDT to induce them to land on the warm, visually conspicuous trap. Two pairs of neighbouring compounds in Kisian village were chosen for this study, each ~100 m apart. Within each pair, approximately 10 cattle were present in one compound and absent in the other. The BG-HDT (excluding tent and pipe used to deliver odours in Experiment 1) was placed next to the corralled cattle herd or in the centre of the compound where cattle were absent. Trapping was performed for six consecutive nights in each pair of compounds between 18:00 h and 07:00 h.

### Experiment 3: Trap validation - does the BG-HDT catch similar abundance and species composition as the original HDT?

In Experiment 3, we tested whether the commercially produced BG-HDT performed as well as the original proof of concept trap used in Hawkes et al. [24], with an additional reference HLC, with respect to mosquito species composition and abundance. We constructed an HDT in accordance with the protocol available at 10.17504/protocols.io.n95dh86. A 3 × 3 Latin Square was conducted in Kisian, comparing HLC, BG-HDT and the original version (O-HDT), both baited with human odour as described in Experiment 1, with the exception that small one-person tents were used. This experiment was completed over 24 nights from May to June 2017.

### Data analysis

Analysis was done using R statistical software version 3.4.1. Data were fitted using Generalized Linear Mixed Effects Statistical Models (GLMMs) to describe effects of collection method on mosquito catches. Since the data were over-dispersed, we used the package *glmmADMB* [34] to fit negative binomial distribution models for the analysis of mosquito numbers. The numbers of female *Anopheles* mosquitoes were assessed as a function of collection method as a fixed effect, and collection sites and days were treated as random effects. A binomial GLM model was used to analyse *Anopheles* species densities per trapping method and a pairwise comparison of means of *Anopheles* species between different trapping methods done by Tukey’s *post-hoc* test.

## Results

Altogether 1807 *Anopheles* and 22,333 culicine mosquitoes were collected in Experiments 1, 2 and 3 combined. Samples collected by HDT were mostly unfed, while HLC yielded the highest proportion of fed *Anopheles* (*n* = 21; 17.10%), whereas there were only 6 blood-fed in HDT-C and none in HDT-H (Table 1). All mosquitoes collected by HDT were in good enough condition for morphological identification, PCR and sporozoite ELISA procedures.

**Table 1:** Numbers of *Anopheles* and culicine species collected by different treatments for each experiment

Experiment	Treatment	*Anopheles* species						Culicine species					
		Fed	Gravid	Half gravid	Unfed	Male	Total	Fed	Gravid	Half gravid	Unfed	Male	Total
Exp. 1 (Kisian, *n* = 24 nights)	HDT-C	1	0	1	1011	0	1013	4	1	1	8610	25	8641
	HDT-H	0	0	1	23	0	24	2	0	1	605	22	630
	HLC	21	0	2	120	5	148	47	6	5	1686	0	1744
Exp. 1 (Homa Bay, *n* = 12 nights)	HDT-C	1	0	0	124	0	125	0	0	0	246	0	246
	HDT-H	0	0	0	9	0	9	0	0	0	26	0	26
	HLC	7	0	1	8	1	16	0	1	6	9	2	18
Exp. 2 (*n* = 6 nights)	Cattle present	41	3	6	86	0	136	570	1	33	2793	1	3398
	Cattle absent	0	0	0	7	0	7	0	0	0	122	1	123
Exp. 3 (*n* = 24 nights)	O-HDT	0	0	0	90	0	90	7	0	0	3089	31	3127
	BG-HDT	1	0	0	119	0	120	2	0	0	2721	9	2732
	HLC	4	0	0	111	4	119	19	32	30	1558	9	1648
Total		76 (4.2)	3 (0.2)	11 (0.6)	1708 (94.5)	10 (0.6)	1807	651 (2.9)	41 (0.2)	76 (0.3)	21,465 (96.1)	100 (0.4)	22,333

### Experiment 1: Comparison of catches from HDTs and HLCs

**Fig. 3:**
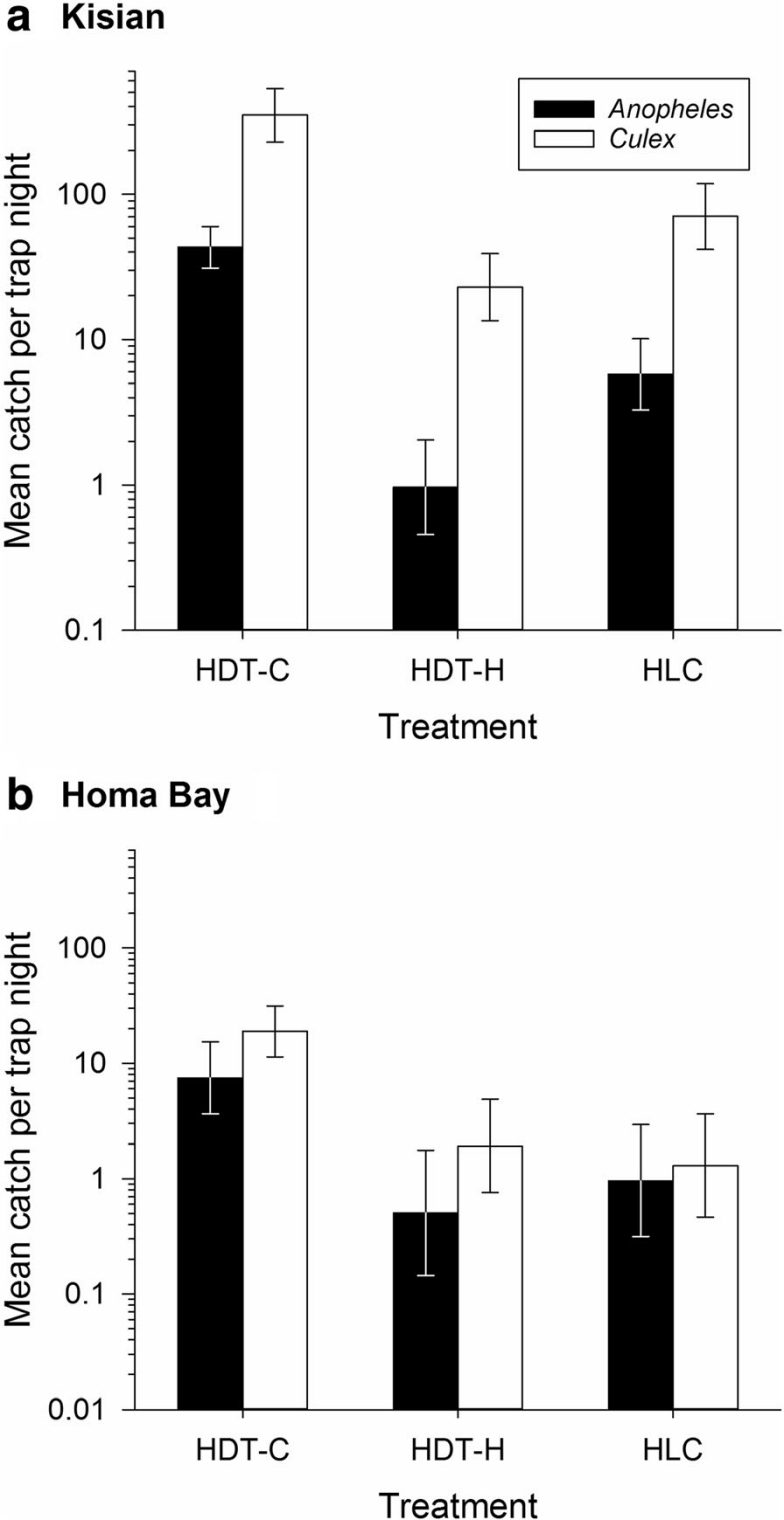
Nightly outdoor catches (mean ± standard error) of *Anopheles* spp. and culicine mosquitoes from cattle-baited HDT (HDT-C), human-baited HDT (HDT-H) and human landing catch (HLC) traps in Kisian (*n* = 24 nights) and Homa Bay (*n* = 12 nights), western Kenya (Experiment 1). Data are plotted on a logarithmic y-axis

Generalized Linear Mixed Effects Statistical Models (GLMMs) was used to all statistical tests. The estimated mosquito abundance in Kisian village differed significantly by trap type. The HDT-C collected a nightly average of 43.2 (95% CI: 26.7–69.8) *Anopheles*, compared to 5.8 (95% CI: 4.1–8.2) in HLC (*z* = -8.99, *P* < 0.001), while HDT-H collected 0.97 (95% CI: 0.4–2.1) per night, significantly fewer *Anopheles* than the HLC (*z* = -6.00, *P* < 0.001). A similar pattern was observed in mean nightly catch of culicine species. These were significantly higher in HDT-C with a mean of 349.6 (95% CI: 208.5–586.3) compared to 70.5 in HLC (95% CI: 46.5–106.7), (*z* = -10.10, *P* < 0.001), while the HDT-H collected 22.9, the fewest culicine mosquitoes (95% CI: 13.6–38.8), significantly less than the HLC (*z* = -7.05, *P* < 0.001; Fig. 3a).

Overall abundance of *Anopheles* in Homa Bay showed a trend of significantly higher numbers of mosquitoes in HDT-C, compared to the other methods. In Homa Bay, a mean of 7.5 (95% CI: 2.8–19.9) *Anopheles* were collected by HDT-C each night, compared to 1.0 (95% CI: 0.4–2.3) in HLC, (*z* = 5.31, *P* < 0.001). No significant difference was found between catches in HLC and HDT-H with a mean of 0.5 (95% CI: 0.1–2.1; *z* = -1.26, *P* = 0.21). As in Kisian, a significantly higher mean number of culicine mosquitoes, 18.9 (95% CI: 7.5–47.3), were also collected by HDT-C each night in Homa Bay, compared to 1.3 (95% CI: 0.7–2.6) in HLC (*z* = 6.61, *P* < 0.001; Fig. 3b).

Both cattle- and human-baited HDTs almost exclusively collected unfed female *Anopheles* (97.4%) while fed *Anopheles* accounted for 17% of HLC samples (Table 1). Sporozoite infection rates were 1.4% (9/635) in HDT-C, 5.5% (1/18) in HDT-H and 0.9% (1/111) in HLC. Sporozoite infection was 0.97% (9/921) in *An. arabiensis* and 1.64% (2/122) in *An. gambiae* (*s.s*.).

**Fig. 4:**
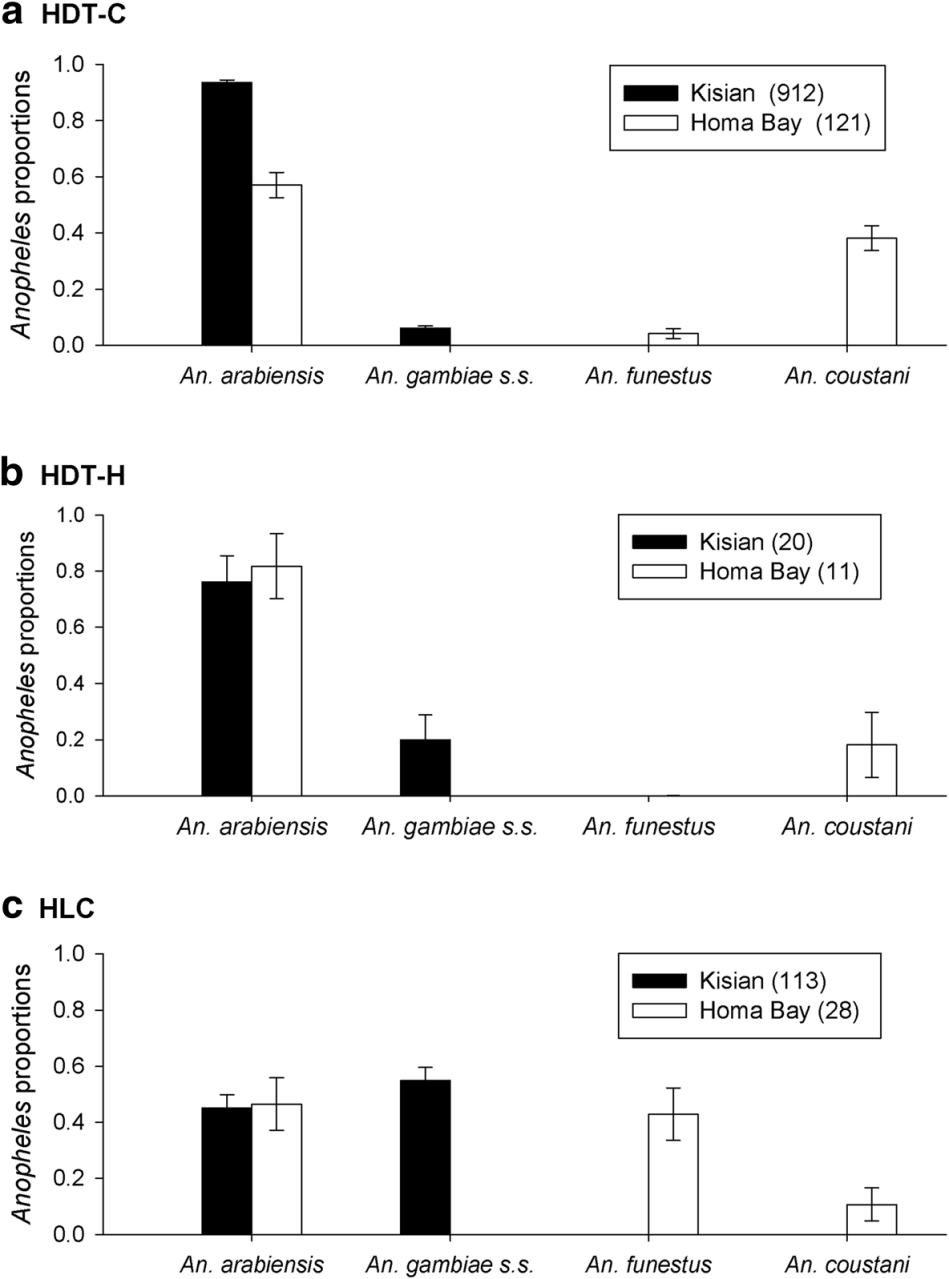
Relative species composition (proportions ± standard error) of *Anopheles* mosquitoes from three outdoor trapping methods [cattle-baited HDT (HDT-C), human-baited HDT (HDT-H) and human landing catch (HLC) traps] in Kisian and Homa Bay, western Kenya (Experiment 1). Numbers in key show total catch of *Anopheles* caught in Kisian (*n* = 24 nights) and Homa Bay (*n* = 12 nights)

Proportions of *Anopheles* species with respect to total anopheline numbers, varied according to trapping method and field location (Fig. 4). From HDT-C collections, *An. arabiensis* comprised the highest proportion of all *Anopheles* species caught in both Kisian (0.94 ± 0.01) and Homa Bay (0.57 ± 0.05). *Anopheles gambiae* (*s.s*.) were collected only in Kisian where they comprised 0.06 ± 0.01 of all anophelines, while both *An. funestus* and *An. coustani* were collected only in Homa Bay at proportions of 0.04 ± 0.02 and 0.38 ± 0.04, respectively (Fig. 4a). Collections by HDT-H were predominantly *An. arabiensis* at both sites (0.76 ± 0.1 of all anophelines in Kisian and 0.82 ± 0.12 in Homa Bay). *An. gambiae* (*s.s*.) comprised 0.20 ± 0.1 of anophelines in Kisian while 0.18 ± 0.12 of anophelines collected in Homa Bay were *An. coustani* (Fig. 4b). Comparable proportions of *An. arabiensis* were collected by HLC in both Kisian and Homa Bay (0.45 ± 0.05 and 0.46 ± 0.09, respectively). The highest proportion of *An. gambiae* (*s.s*.), was observed in HLC collections in Kisian, where it made up 0.55 ± 0.05 of all anophelines, while *An. funestus* comprised 0.43 ± 0.09 of all anophelines collected in Homa Bay (Fig. 4c).

In Kisian, significantly higher proportions of *An. arabiensis* were found in HDT-C compared to HDT-H (*z* = -2.8; *P* = 0.01), and in HDT-H compared to HLC (*z* = -2.5; *P* = 0.03). A significantly lower proportion of *An. arabiensis* was observed in HLC compared to HDT-C (*z* = -12.4; *P* < 0.001). Significantly higher proportions of *An. gambiae* (*s.s*.) were observed in HLC compared to HDT-C (*z* = 12.5; *P* < 0.001), HLC compared to HDT-H (*z* = 2.7; *P* = 0.02) and HDT-H compared to HDT-C (*z* = 2.3; *P* = 0.05). Only two *An. funestus* were collected by HDT-C in Kisian, hence no analysis was performed on this species.

In Homa Bay, there was no significant difference in the proportion of *An. arabiensis* caught by the different collection methods. Significantly higher proportions of *An. funestus* were collected in the HLC compared to HDT-C (*z* = 4.8; *P* < 0.001). No *An. funestus* were collected by HDT-H. *An. coustani* was sampled by all collection methods. HDT-C collected significantly higher proportions of *An. coustani* compared to HLC (*z* = -2.66; *P* = 0.03), while no significant differences were found between HDT-C and HDT-H or between HLC and HDT-H.

### Experiment 2: Catches from unbaited HDT

**Fig. 5:**
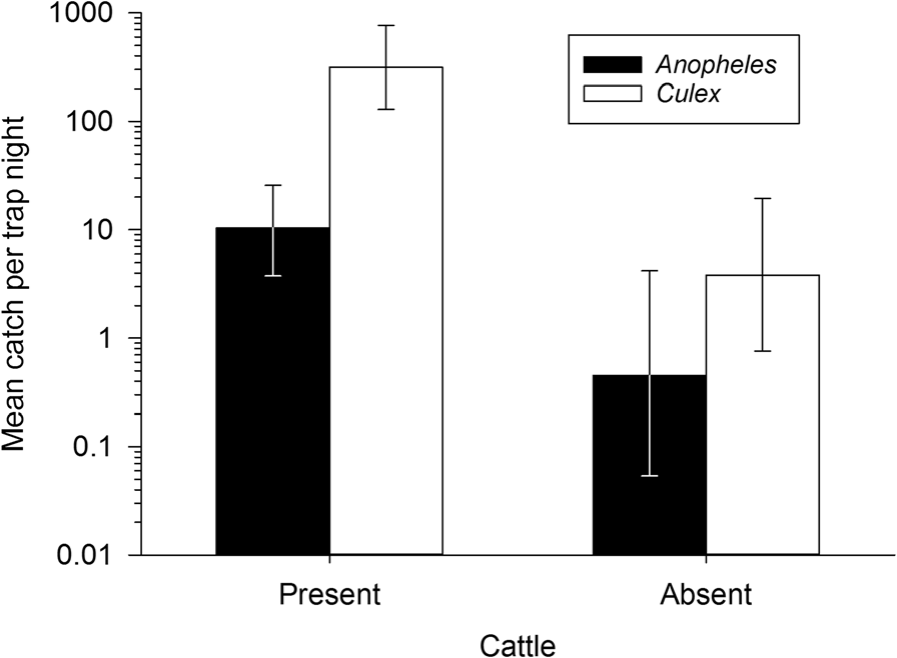
Comparison of mean (± standard error) catches by Host Decoy Traps in the presence or absence of cattle in Kisian, western Kenya. Mean nightly outdoor catch (*n* = 6 nights/site for each treatment) of *Anopheles* spp. and culicine mosquitoes (Experiment 2). Data are plotted on a logarithmic y-axis

Unbaited BG-HDTs placed either next to a herd of corralled cattle or in a compound with no cattle present captured *Anopheles* mosquitoes. The traps collected mostly *An. arabiensis* at proportions of 0.97 ± 0.02 and 0.8 ± 0.2 in the presence and absence of cattle, respectively. These differences were not statistically significant. However, the HDT collected a mean of 10.4 (95% CI: 2.0–55.0) *Anopheles* each night in the presence of cattle *versus* 0.45 (95% CI: 0.1–1.7) when cattle were absent (*z* = -3.81; *P* = 0.0001). A significantly higher mean number of culicine mosquitoes were collected in the presence of cattle, 314.5 (95% CI: 70.0–1412.3) *versus* 3.83 (95% CI: 1.4–10.5) in compounds without cattle (*z* = -6.92, *P* < 0.001; Fig. 5). No sporozoite-positive *Anopheles* were detected in Experiment 2, however 30% of *Anopheles* mosquitoes in the HDT next to cattle were blood-fed, which may reflect partial blood meals on the available cattle (Table 1).

### Experiment 3: Trap validation - does the BG-HDT catch similar abundance and species composition as the original trap?

**Fig. 6:**
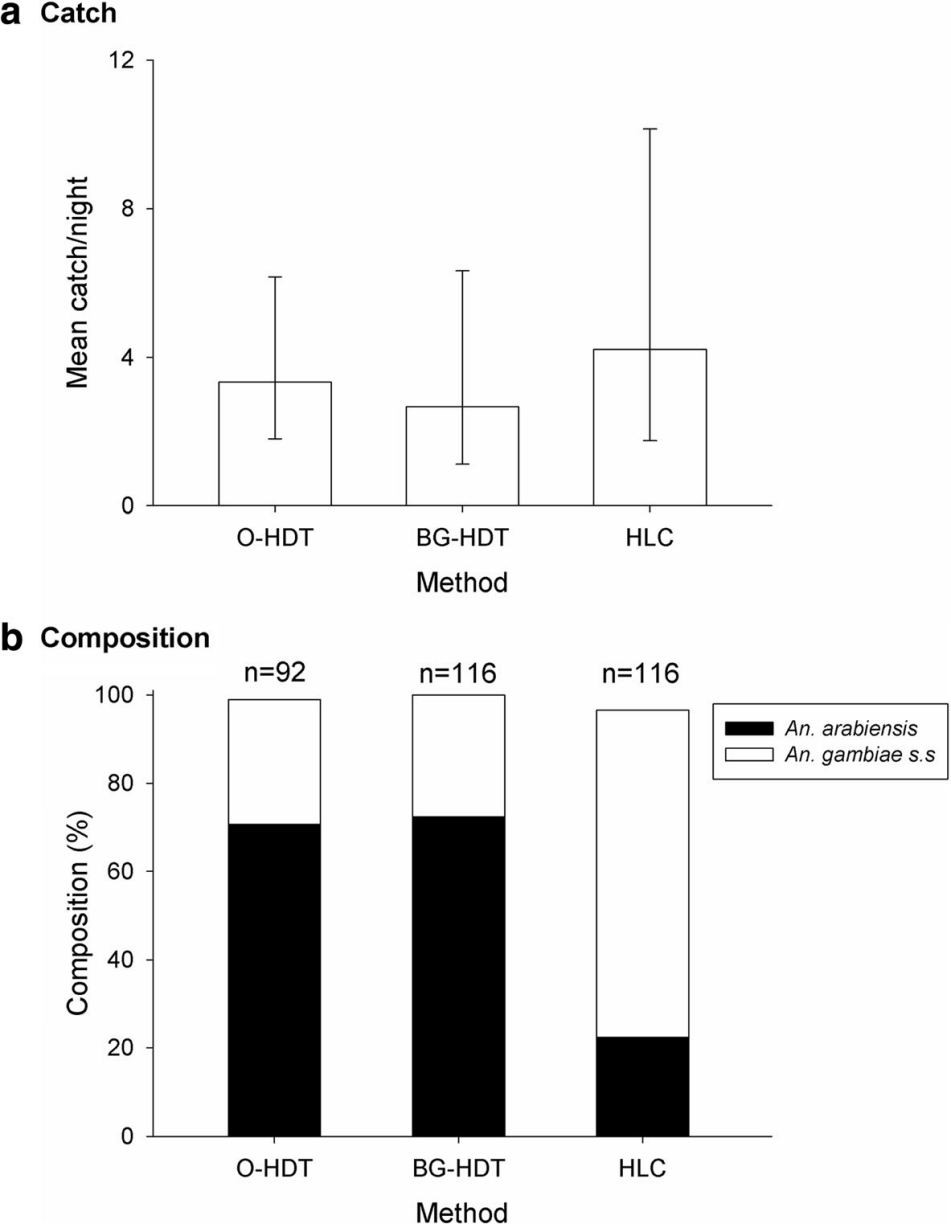
Nightly outdoor catches (mean ± SE; *n* = 24 nights) of *Anopheles* mosquitoes with the original Host Decoy Trap (O-HDT), the BG-HDT and the human landing catch (HLC), in Kisian, western Kenya (Experiment 3)

We compared the commercial BG-HDT produced by Biogents and the O-HDT, the original proof of concept version, alongside a standard HLC. We found no significant difference (*z* = -0.73; *P* = 0.46) in the mean nightly outdoor catch of *Anopheles* between the commercial BG-HDT, which caught 3.33 (95% CI: 1.4–8.0), and the original version made using locally available materials, which caught 2.66 (95% CI: 1.1–6.5) per night (Fig. 6). There was also no significant difference in mean nightly *Anopheles* catch between the commercial BG-HDT and HLC [4.21 (95% CI: 2.2–7.9); *z* = -0.74; *P* = 0.46]. The commercial BG-HDT and O-HDT caught near identical proportions of *An. arabiensis* (72% and 69% of specimens, respectively; *z* = -0.50; *P* = 0.86).

## Discussion

Our results demonstrate that the HDT baited with cattle odour is a highly efficient method of sampling outdoor biting anophelines, with a cattle-baited HDT catching consistently more *Anopheles*, mainly *An. arabiensis*, than the HLC. Overall, the cattle-baited HDT caught over seven times more *Anopheles* than HLC outdoors. There were also significant differences in the species composition captured by traps baited with different hosts. This result suggests that HDTs may be useful both for collecting large numbers of mosquitoes outdoors, as well as for elucidating mosquito host choice. Our ability to trap mosquitoes when placed in the presence of cattle outdoors demonstrates how the HDT could be deployed as a passive monitoring device for use in outdoor peri-domestic environments. The HDT incorporates sensory stimuli used by host biting mosquitoes to locate their next blood meal and represents a potential new tool for sampling host-seeking mosquitoes, particularly in outdoor environments. The high proportion of unfed *Anopheles* in HDT collections demonstrate that it is an exposure-free trap. Comparatively, high blood-fed rate in HLC is likely a reflection of potential blood meal from the collectors who are at the risk of potentially infectious mosquito bites. We recommend further improvement of the trap with development of artificial lures that mimic a full arrange of host-associated odours to be used in combination with other mosquito host stimuli for malaria vector surveillance.

The number of *Anopheles* caught in HDT-H was significantly lower than HLC in the Kisian experiment while no significant difference was observed between the two methods in Homa Bay. In the initial development of the trap, HDT-H caught significantly more *Anopheles* overall than the HLC [24]. In the present study, local vector populations are composed of *An. gambiae* (*s.s*.), *An. arabiensis*, *An. funestus* and *An. coustani*, whereas *An. coluzzii* is predominant in the area of Burkina Faso where the first evaluation of HDT took place. Given that Experiment 3 confirmed the original prototype used in Burkina Faso [24] showed similar catch abundance and composition to the BG-HDT deployed in Experiments 1 and 2, the observed difference in HDT performance is likely a result of species behavioural differences rather than differences in trapping method. Measurement of CO_2_ between the HDTs showed that 2.44 times more CO_2_ was released from the HDT-C tent than the HDT-H tent. However, there were ~44 times more *Anopheles* and ~14 times more culicines in the HDT-C than in the HDT-H. The effect of differing CO_2_ concentrations released from the cattle and human tents on the respective HDT catches demonstrates that there is a non-linear relationship between CO_2_ and attractiveness to mosquitoes, which merits further research.

*Anopheles arabiensis* was the predominant species in catches by HDT-C, highlighting the behavior of this species with reference to feeding location and host choice. Previous studies in western Kenya have largely associated *An. arabiensis* with cattle feeding, and outdoor biting with occasional feeds on humans both indoors and outdoors [2, 26, 27, 35]. Even though the overall catch of *An. arabiensis* was low in both HDT-H and HLC, the vector species comprised a high proportion of *Anopheles* trapped by the two methods at both sites with some captured mosquitoes having sporozoite infection, indicating previous feeding on humans, although likely at lower rates than *An. gambiae* or *An. funestus*. Earlier investigations of *An. arabiensis* biting behavior in western Kenya found that outdoor resting *An. arabiensis* did not feed on humans, whereas those caught resting indoors had a human blood index (HBI) of 0.23 [27]. In northern Tanzania, 90.3% of *An. arabiensis* were captured in traps baited with cattle odour compared to 9.7% which were attracted to human odour [36]. In Ethiopia, an evaluation of blood-feeding behavior of *An. arabiensis* using host-baited sampling methods showed that this species fed preferentially on humans over cattle outdoors, but with a preference for cattle-biting outdoors over human-biting indoors [25, 37]. These studies illustrate the diversity of feeding behaviour of *An. arabiensis*, which makes this species particularly difficult to control by LLINs and IRS.

Human-baited traps, HDT-H and HLC caught the largest proportions of *An*. *gambiae* (*s.s*.). While earlier studies investigating host selection reported the species to feed more frequently on humans indoors [2, 26, 27, 35], there is a recent report of an unusually high frequency of animal and mixed blood meals in *An. gambiae* (*s.s*.) [10] and a shift in biting time [38] in regions of western Kenya highlands with high bednet coverage. These observations suggest possible behavioral modification in the presence of bednets. While our data are unable to confirm any of these observations, we recommend further studies to determine current contribution of *An. gambiae* (*s.s*.) to malaria transmission both indoors and outdoors in the lake endemic regions of western Kenya, following previous reports of historical population decline of the species associated with the introduction of bednets [3].

Additional control tools that target outdoor-biting vector populations are needed to supplement LLINs and IRS [7, 39]. Zooprophylaxis by keeping cattle around houses has been suggested as a strategy to protect humans from malaria [36]. Classical zooprophylaxis (without insecticides) may not have a significant impact on the malaria vectorial capacity of *An. arabiensis* [37] in regions where the vector bites both humans and cattle. Indeed, the presence of cattle may result in the proliferation of the species and sustain outdoor transmission. However, treating cattle with insecticides or endectocides, such as ivermectin, may be a viable strategy [40]. Recent evaluation of endectocide administration to local Zebu cattle under semi-field conditions in western Kenya showed a significant reduction in survival of *An. arabiensis* of up to 21 days post-treatment [41]. Furthermore, a field evaluation of topical formulations of eprinomectin against *An. arabiensis* in western Kenya showed a 38% reduction in indoor resting densities of the species within one-week post-treatment [42]. The HDT is suitable for sampling outdoor-biting vectors under such treatments, and therefore, could be a valuable method for monitoring the impact of the next generation of control interventions that target malaria vectors, including periodic assessment of host preference. The numbers of *An. arabiensis* collected and killed each night by the HDT also raises the question of whether the concept of host decoys can be developed as a behaviour-based vector control tool, similar to the Suna trap [18] or to the lethal targets used to lure and kill tsetse vectors of trypanosomes [43].

## Conclusions

The HDT, which combines odours, heat and a visually-conspicuous stimulus to simulate a host, provides the basis of a system to sample *Anopheles* mosquitoes, particularly outdoor feeding mosquitoes that tend to feed primarily on other hosts but may be involved in residual transmission of malaria. The cattle-baited HDT is particularly effective for *An. arabiensis*, an important vector of malaria which feeds, in part, outdoors on cattle and is, therefore, not efficiently sampled or controlled by standard methods. The HDT offers the prospect of a system to monitor and potentially control *An. arabiensis* and other outdoor-biting mosquitoes more effectively. To achieve a practical, standardized system, the use of artificial host odours to replace the natural odours used in this and previous studies of the HDT should be explored.

## Abbreviations

BG-HDT: Host decoy trap manufactured by Biogents AG
CDC: Centers for disease control and prevention
ELISA: Enzyme-linked immunosorbent assay
GLMM: Generalized linear mixed effects statistical models
HBI: Human blood index
HDT: Host decoy trap
HDT-C: Host decoy trap - cow-baited
HDT-H: Host decoy trap - human-baited
HLC: Human landing catch
IRS: Indoor residual spraying
KEMRI/SERU: Kenya Medical Research Institute/ Scientific and Ethics Review Unit
LLIN: Long-lasting insecticidal net
O-HDT: Host decoy trap - original construction
PCR: Polymerase chain reaction
PVC: Polyvinyl chloride
